# Melanin Bleaching and Melanogenesis Inhibition Effects of *Pediococcus acidilactici* PMC48 Isolated from Korean Perilla Leaf Kimchi

**DOI:** 10.4014/jmb.2003.03007

**Published:** 2020-04-07

**Authors:** Sukyung Kim, Hoonhee Seo, Hafij Al Mahmud, Md Imtiazul Islam, Omme Fatema Sultana, Youngkyoung Lee, Minhee Kim, Ho-Yeon Song

**Affiliations:** 1 Department of Microbiology and Immunology, School of Medicine, Soonchunhyang University, Cheonan 31151, Republic of Korea; 2 Probiotics Microbiome Convergence Center, Asan 31538, Republic of Korea; 3 Emory university, Institute for Quantitative Theory and Methods (QuanTM), GA 30322, USA

**Keywords:** *Pediococcus acidilactici* PMC48, melanin, tyrosinase, perilla leaf kimchi

## Abstract

Overproduction and accumulation of melanin in the skin will darken the skin and cause skin disorders. So far, components that can inhibit tyrosinase, a melanin synthase of melanocytes, have been developed and used as ingredients of cosmetics or pharmaceutical products. However, most of existing substances can only inhibit the biosynthesis of melanin while melanin that is already synthesized and deposited is not directly decomposed. Thus, their effects in decreasing melanin concentration in the skin are weak. To overcome the limitation of existing therapeutic agents, we started to develop a substance that could directly biodegrade melanin. We screened traditional fermented food microorganisms for their abilities to direct biodegrade melanin. As a result, we found that a kimchi-derived *Pediococcus acidilactici* PMC48 had a direct melanin-degrading effect. This PMC48 strain is a new strain, different from *P. acidilactici* strains reported so far. It not only directly degrades melanin, but also has tyrosinase-inhibiting effect. It has a direct melanin- decomposition effect. It exceeds existing melanin synthesis-inhibiting technology. It is expected to be of high value as a raw material for melanin degradation drugs and cosmetics.

## Introduction

Melanin is primarily an indole derivative of L-dihydroxyphenylalanine. It is highly oxidative in nature. It is the major pigment present in surface structures of vertebrates [1]. The name “melanin” comes from the ancient Greek melanos, meaning “dark”. The origin of its name is currently unclear. However, it is usually attributed to a Swedish chemist Berzelius (1840) [2]. Human skin coloration is dependent almost exclusively on the concentration and spatial distribution of chromophores melanin and haemoglobin, where melanin plays a dominant role in driving constitutive coloration [3]. Skin darkening is due to the presence of a chemically inert and stable pigment known as melanin that is produced deep inside the skin but is displayed as a mosaic at the surface of the body [4].

It has been traditionally believed that skin pigmentation is the most important photoprotective factor because melanin not only functions as a broadband UV absorbent, but also possesses antioxidant and radical scavenging properties. Although moderate amounts of melanin have good effects on the human body, deposition by excessive synthesis can cause pigmentary disorders such as lentigo, naevus, freckles, age spots, chloasma, and melanoma [5]. An increased amount of melanin in the skin is called hypermelanosis or melanoderma [6]. Hypermelanosis in the epidermis is caused by an increase in melanin in basal and suprabasal layers of the skin associated with a normal or elevated amount of melanocytes. This is a common dermatologic problem that may have substantial impacts on the patient since it affects the appearance and quality of life [7], Treatment of hypermelanosis involves the use of topical hypopigmenting agents such as hydroquinone, tretinoin, kojic acid, azelaic acid, and arbutin that can inhibit novel synthesis of melanin in melanocytes [8]. Most previous treatment options for these disorders remain unsatisfactory [9]. Therefore, it is necessary to develop a hypermelanosis treatment agent having a new mechanism of action different from existing treatments.

An alternative way of skin lightening is by decolouring melanin pigment. Although melanins are very stable compounds, under special conditions chemical or photochemical degradation and biodegradation by fungi are possible [10]. It has been reported that *Aspergillus fumigatus* and Geotrichum, Stropharia, Geotrichum, and Sporotrichum gena found in contaminated areas have melanin degradation effects [10-12]. As described above, unlike existing melanin synthesis inhibitors, melanin-decomposing microorganisms are expected to be of great value as therapeutic agents for hypermelanosis with new mechanisms of action.

This research is about the development of a new microorganism that can directly degrade melanin that has already been synthesized and deposited while having the function of inhibiting melanin synthesis. The ultimate purpose of this study is to develop medicines and cosmetics using the new microorganism. Therefore, microorganisms derived from traditional Korean fermented foods were screened. Such foods have been consumed in Korea for a long time with secured safety.

## Materials and Methods

### Isolation of Melanin Degrading Microorganisms from Korean Traditional Fermented Foods

Twenty kinds of traditional fermented foods were obtained from various parts of Korea and microorganisms were isolated. Abalone and sea urchin sauce, conche & ghee sauce, cured cheese, cured kimchi, cuttle fish sauce, kimchi (fresh), kimchi (old), seasoning soybean paste, mustard pickles, soypaste mixed with red peppers, perilla leaf kimchi, and soybean sauce were used for isolating different kinds of microorganisms. Several kinds of media were used and aerobic/anaerobic condition were given for each medium to isolate 252 types of isolates. Brain Heart Infusion (BD, 211065), M17 (Kisanbio, MB-M1192), Tos-MUP (Kisanbio, MB-T0892), and MRS (BD, 288210) agar were used for this experiment.

### Agar Well Diffusion Method

The agar well diffusion method was applied to measure melanin degradation. Briefly, 100 μl of culture of *P. acidilactici* PMC48 was added into modified agar containing 0.2 mg/ml melanin (Sigma Chemical Co., USA). Arbutin and hydroquinone at 20 mM were used as controls. After 24 h of incubation, clear zone nearby each hole was checked.

Melanin degradation assay in broth. The tube broth method was applied to measure melanin degradation. Briefly, 100 μl of culture and culture filtrate of *P. acidilactici* PMC48 were added into 10 ml of modified broth containing 0.2 mg/ml melanin (Sigma Chemical Co.). Arbutin and hydroquinone at 20 mM were used as controls. After 72 h of incubation at 37oC with shaking (120 rpm), melanin amount was checked visually after centrifugation.

### Tyrosinase Inhibition Test Using Tyrosinase as a Substrate

In order to assay the inhibitory effect of *P. acidilactic*i culture filtrate on mushroom tyrosinase, dose-dependent inhibition experiments were carried out in triplicate. In brief, 10 μl of an aqueous solution of mushroom tyrosinase (2,000 U/ml) (Sigma Chemical Co.) in 0.05 M phosphate buffer was added to a 96-well microplate in a total volume of a 270 μl mixture containing 40 μl of 1.5 mM L-tyrosine solution, and 230 μl of 100 mM phosphate buffer (pH 6.8). A sample solution (20 μl) was added to the reaction mixture (280 μl) and incubated at 37°C for 60 min. Following incubation, the amount of L-DOPA produced in the reaction mixture was determined spectrophotometrically at 490 nm (OD490) with a microplate reader. Tyrosinase activity (%) was calculated using the following equation: {[(Sample + tyrosinase) - (sample alone)] / (tyrosinase)}×100.

### Tyrosinase Inhibition Test based on L-DOPA

In order to assay the inhibitory effect of *P. acidilactici* PMC48 culture filtrate on mushroom tyrosinase, dose- dependent inhibition experiments were carried out in triplicate as described previously with a minor modification [29]. In brief, 15 μl of an aqueous solution of mushroom tyrosinase (2,000 U/ml) (Sigma Chemical Co.) was added to a 96-well microplate in a total volume of a 270 μl mixture containing 255 μl of 0.1 M sodium phosphate buffer solution, 15 μl of *P. acidilactici* PMC48 culture filtrate. The assay mixture was incubated at 37oC for 30 min. Following incubation, 15 μl of 10 mM L-DOPA (Sigma Chemical Co.) was added additionally into 96- well plate. The amount of dopachrome produced in the reaction mixture was determined spectrophotometrically at 490 nm (OD490) with a microplate reader. Tyrosinase activity (%) was calculated using the following equation: {[(Sample + tyrosinase) - (sample alone)] / (tyrosinase)}×100.

### DPPH Radical Scavenging Effect Test

The purple color of DPPH solution fades rapidly after interaction with proton-radical scavengers. The radical scavenging activity of *P. acidilactici* PMC48 was determined according to a previous report. Different concentrations (1.56% to 100%) of *P. acidilactici* PMC48 (20 μl) were mixed with 100 mM Tris-HCl buffer (80 μl, pH 7.4) and then added to 100 μl of 100 μM DPPH in ethanol (final concentration 50 μM). After vigorous shaking, the mixture was left in the dark at room temperature for 30 min. The absorbance of the resulting solution was measured spectrophotometrically at 517 nm. DPPH radical scavenging activity was expressed as percentage of the control (0% *P. acidilactici* PMC48).

### Melanin Content Measurement

B16F10 (*Mus musculus* skin melanoma) cells were obtained from the Korean Cell Line Bank (KCLB, Korea). These cells were cultured in Dulbecco’s Modified Eagle’s Medium (GIBCO, USA) supplemented with 2 mM L- glutamine, 10% heat-inactivated fetal bovine serum (GIBCO), and 1% penicillin-streptomycin at 37°C in fully humidified air with 5% CO_2_ and subcultured twice weekly. In the current study, melanin content was used as an index of melanogenesis. Estimations of melanin content were performed using a modified method of Bilodeau *et al*. (2001). In short, B16F10 cells (5*10^4) were plated onto 6-well dishes and incubated in the presence of 100 nM α-MSH for 24 h. Cells were then incubated for 72 h with *P. acidilactici* PMC48 culture filtrate at concentrations of 3.12% and arbutin at 1 or 2 mM. After washing twice with PBS, samples were dissolved in 100 μl of 1N NaOH. These samples were then incubated at 60oC for 1 h and mixed to solubilize melanin. Absorbance at 405 nm was compared with a standard curve of synthetic melanin.

### API 50 CHL Test

Fermentation of carbohydrates was determined using API 50 CHL, a standardized system consisting of 50 biochemical tests for the study of carbohydrate metabolism by microorganisms. Pure water (10 ml) was dispensed into the incubation box with the strip placed in the incubation box after bacterial cultures were introduced into the API 50 CHL system in API 50 CHL medium (5 ml) in concentration 2 McFarland. The set-up system was then incubated at 37°C for 48 h after wells were filled with bacterial suspensions by the line mark with the addition of mineral oil. Identification tables were prepared as (+/-) according to color change in evaluation of results of API strips reaction. Numerical profiles of strains were identified adding positive values in indicative table. Species designations were identified by evaluating with an identification software apiweb^TM^.

### Whole Genome Sequencing

Genomic DNA of *P. acidilactici* PMC48 was extracted using QIAamp DNA Mini kit (Qiagen, Germany). Sequencing analysis was performed in Chunlab, Inc (Korea). PacBio sequencing data were assembled with PacBio SMRT Analysis 2.3.0 using the HGAP2 protocol (Pacific Biosciences, USA). Resulting contigs from PacBio sequencing data were circularized using Circlator 1.4.0 (Sanger institute, UK). Sequence reads and assemblies are deposited in the National Center for Biotechnology Information (NCBI) database under accession number PRJNA612145.

### Cell Cytotoxicity

Cell viability assay of B16F10 was performed by using 3-(4,5-dimethylthiazol-2-yl)-2,5-diphenyltetrazolium bromide (MTT) [30]. Briefly, 1* 10^4 cells/well was seeded into a 96-well plate. These cells were exposed to *P. acidilactici* culture filtrate (1.56, 3.12, 6.25, and 12.5%) for 24 h. Then MTT solution was added to each well. The insoluble derivative of MTT produced by intracellular dehydrogenase was solubilized with ethanol-DMSO (1:1 mixture solution). The absorbance of each well at 570 nm was read using a microplate reader. The amount of MTT in bacterial culture filtrate treated group was compared to that of the control group. The higher relative amount of MTT measured indicated that the culture filtrate was not cytotoxic to B16F10 cells.

## Results and Discussion

### Isolation of Melanin Degrading Microorganisms from Korean Traditional Fermented Foods

Twenty kinds of traditional fermented foods were obtained from various parts of Korea and microorganisms were isolated. A total of 252 types of isolates were grown using MRS agar and 49 kinds of microorganisms were harvested, except for overlapping isolates by product and morphology. Microorganisms that could degrade melanin were screened using the agar well diffusion method with melanin-containing agar media. Among 49 kinds of microorganisms, isolate PMC48 originated from perilla leaf kimchi degraded melanin around its pellets to form a clear zone. Genome analysis and melanin degradation or biosynthesis inhibition effects of the isolated microorganism, PMC48, was further performed or analyzed in depth.

### Biochemical Characteristics of the Isolated Bacterial Strain

Current methods for characterizing and identifying bacterial isolates include a variety of routine phenotypic, biochemical, enzymatic, and molecular tests. The use of phenotypic and biochemical tests for identification has been the traditional standard for many years [13]. Biochemical characterization of isolated bacterial strains was carried out for identification and phenotypic characterizations of bacteria (Table 1). Based on biochemical and morphological tests according to the Bergey’s manual [14], PMC48 isolate was identified as *Lactobacillus brevis*. Its phenotypic characteristics alone were insufficient to differentiate it from other bacterial isolates due to the standardization of conventional methods [15]. It has also been reported that phenotypic characterization results cannot be used for direct comparison because these results require full background knowledge for each test [15]. Furthermore, 16S rRNA sequence analysis was used to ensure accurate taxonomic position of metal-resistant bacteria reported in this study.

### Identification of Isolated Bacterial Strains based on 16S rRNA Gene Sequence Analysis

The use of 16S rRNA gene sequences to study bacterial phylogeny and taxonomy has been by far the most common housekeeping genetic marker used [16]. Therefore, 16S ribosomal RNA sequences have been used extensively in the classification and identification of bacteria [17]. The comparison of almost complete 16S rRNA gene sequences has been widely used to establish taxonomic relationships between prokaryotic strains. Sequence similarity of 98.65% is currently recognized as the cutoff for delineating species [17]. PMC48, a melanin- degrading isolate, was identified taxonomically by robust method of 16S rRNA gene sequencing (Table 2). By comparing its 16S rRNA sequence with those deposited at The National Center for Biotechnology Information (NCBI) reference sequence database, the isolated strain was found to belong to *Pediococcus acidilactici*. This isolate shared more than 99% sequence similarities with its closest relative. This strain shared significant similarity (99.47%) with *P. acidilactici* DSM 20284. However, many investigators have found that 16S rRNA gene sequencing data have resolution problems at genus and/or species level [16]. Therefore, we performed additional genome analysis for exact species determination with in-depth genetic analysis of selected strain.

### Study of Genome Properties and Comparative Analysis of the Selected Isolate, PMC48

**Fig. 1:**
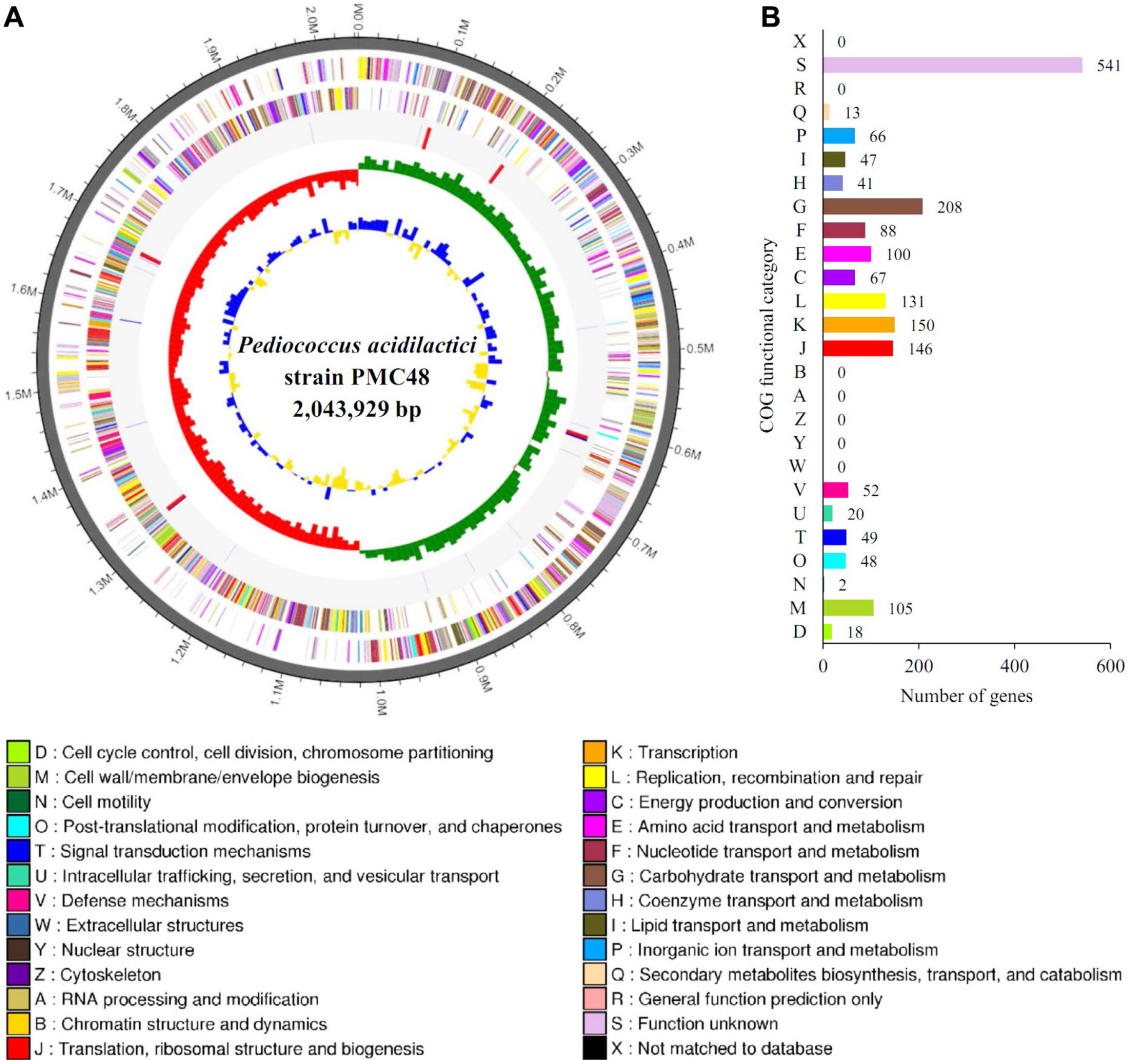
High-throughput genome sequencing of *Pediococcus acidilactici* strain PMC48. (**A**) Circularmap of *Pediococcus acidilactici* PMC48 strain genome. Antisense and sense strands (colored according to COG categories) and RNA genes (red, tRNA; blue, rRNA) are shown from the outer periphery to the center. Inner circles show the GC skew, with yellow and blue indicating positive and negative values, respectively, and the GC content is indicated in red and green. This genome map was visualized using CLgenomics. (**B**) Relative abundance of cluster of orthologous groups (COG) functional categories of genes.

Primary features of the genome of strain PMC48 are presented in Fig. 1. Strain PMC 48 contained a single, circular chromosome of 2,043,929 bp, with an average GC content of 42.2%. We detected 2,026 coding sequences (CDSs) in the genome, with an average length of 870.5bp (Fig. 1A). As shown in Fig. 1B, predicted CDSs were grouped by Clusters of Orthologous Groups (COG) functional categorizations. Among these CDSs, 1,892 proteins were assigned to COG families [18]. Biological functions could be defined for 1,351 (66.7%) of predicted proteins, while 541 CDSs (26.7%) were homologous to conserved proteins with unknown functions in other organisms. The remaining 134 hypothetical proteins (6.6%) had no match with any known proteins in the database. Furthermore, 57 tRNA and 15 rRNA genes were predicted.

**Fig. 2:**
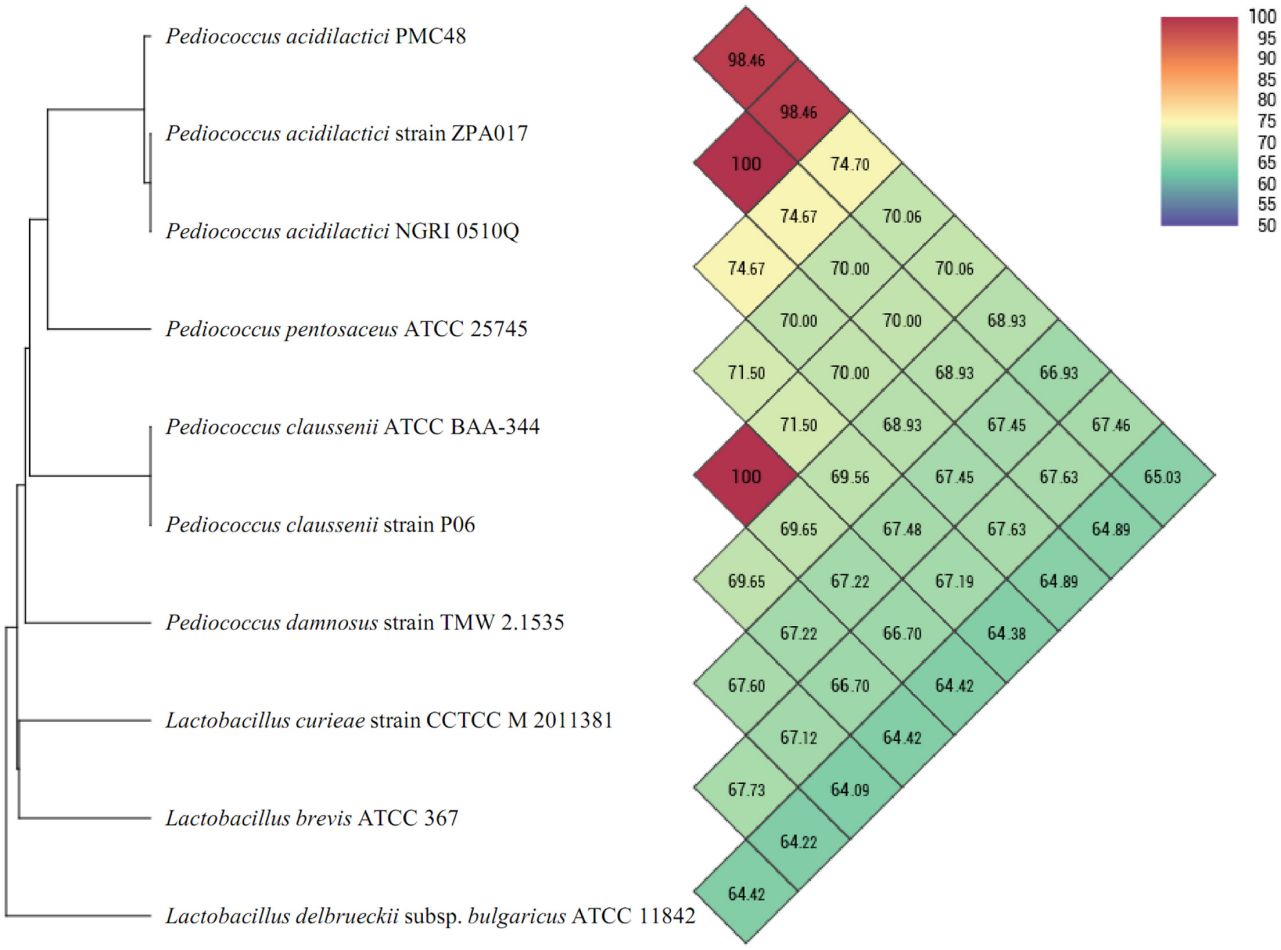
Phylogenomic tree and OrthoANI result calculated with available genomes of *Pediococcus* and *Lactobacillus* species. Values bigger than 96% indicate that strains belong to the same species. The results between two strains are given in the junction point of the diagonals departing from each strain, *i.e.*, OrthoANI value between *Pediococcus acidilactici* PMC48 and *Lactobacillus delbrueckii* subsp. bulgaricus ATCC 11842 is 74.0%. (2-column ﬁtting image).

OrthoANI provides a more robust and faster means of calculating average nucleotide identity for taxonomic purposes [19]. Using whole genome sequence data of PMC48 strain, similarity analysis was performed using the OrthoANI method with strains that shared high similarities in 16S rRNA analyses (Fig. 2). When OrthoANI analysis was performed to compare the isolate identified from this study to all publicly available *P. acidilactici* genomes, similarities were 98.46% for other *P. acidilactici* strains (ZPA017, NGRI 0510Q), which was significantly above the cut-off value of 95% for species delineation [20]. When our isolate was compared to non- acidilactici *Pediococcus* spp. genomes, identities values were below 80%. In addition, identities values with *Lactobacillus brevis* based on biochemical results and *Lactobacillus* species which showed low similarity in 16S rRNA analysis were significantly lower. These results strongly suggest that the melanin-degrading strain, PMC48 strain, is *P. acidilactici*.

We then compared *P. acidilactici* PMC48 genome information and publicly available genome information of other strains of *P. acidicactici* (LPBC161 [21], S1 [22], MA18/5M [23], K3 [24], NGRI 0510Q [25]) (Table 3). Although they were the same species, their genome sizes, GC contents, and numbers of CDS, rRNA, and tRNA were all different. This finding shows that PMC48 is a new strain of *P. acidilactici*. The draft genome sequence of strain PMC48 will further help us understand its melanin-degrading potential at genetic level.

### Melanolytic Activity of *P. acidilactici* PMC48

**Fig. 3:**
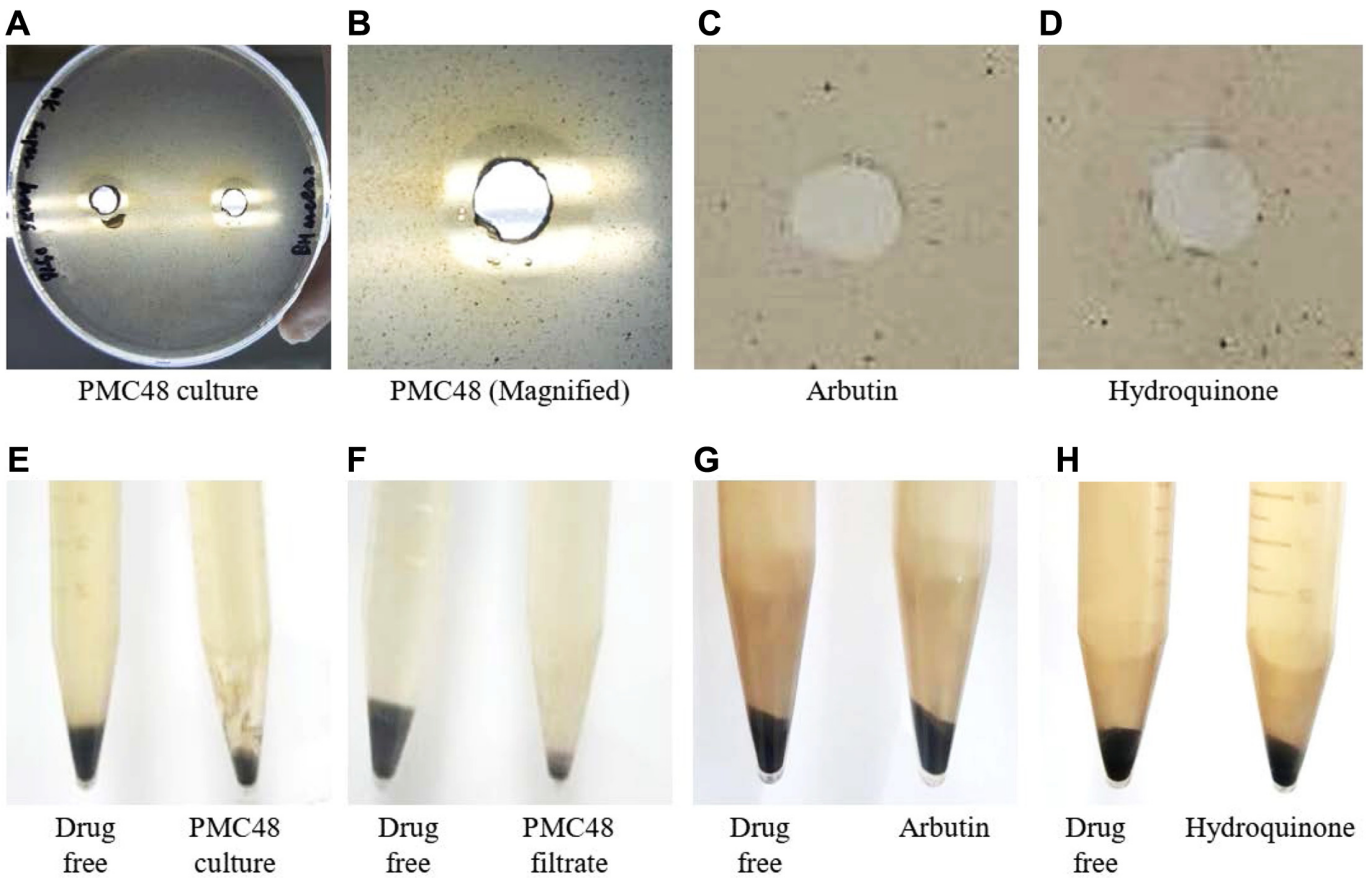
Degradation profiles of melanin by PMC48 strain. The agar well diffusion method (**A**-**D**) and the tube broth method (**E**-**H**) were applied to measure melanin degradation. The melanin degradation effect of the broth culture (**A**, **B**, **E**) or cell free culture filtrate (**F**) of PMC48 was measured. PMC48 culture of late exponential phase was used, and the filtrate was prepared by filtering the supernatant from which cells were removed by centrifugation of the culture medium. Control drugs, arbutin (**C**, **G**) and hydroquinone (**D**, **H**) were tested under the same conditions. In the agar well diffusion method, agar medium containing 1 mg/ml of melanin was used, and 100 μl of culture medium of PMC48 or same amount of control drugs (10 mg/ml of stock solution) were added and they were incubated for 24 h. In the tube broth method, a 10 ml liquid medium containing 1 mg/ml of melanin was used, and 100 ul of PMC48 culture solution and culture filtrate or final control 1 mM control drugs were added to the tube and after 24 h incubation and melanin was extracted.

Melanin-degrading activity of *P. acidilactici* PMC48 isolated from perilla leaf kimchi was measured (Fig. 3). Using the agar well diffusion method, a clear zone was formed around the PMC48 cell culture, indicating that the strain could directly degrade melanin (Fig. 3A). Furthermore, PMC48 strain’s culture filtrate also formed a clear zone, with size similar to the melanin degradation effect by the bacterial cell culture (Fig. 3B). Under the same conditions, arbutin or hydroquinone did not form a clear zone (Figs. 3C and 3D). Using the tube broth method, PMC48 culture degraded melanin. It could be clearly seen that melanin was reduced (Fig. 3E). Cell free culture filtrate of PMC48 also degraded melanin (Fig. 3F). Under the same conditions, arbutin or hydroquinone did not show melanin degrading effect (Figs. 3G and 3H). These results strongly suggest that PMC48 strain has an excellent effect of directly degrading melanin.

### Inhibitory Effect of *P. acidilactici* PMC48 on Melanin Biosynthesis

**Fig. 4:**
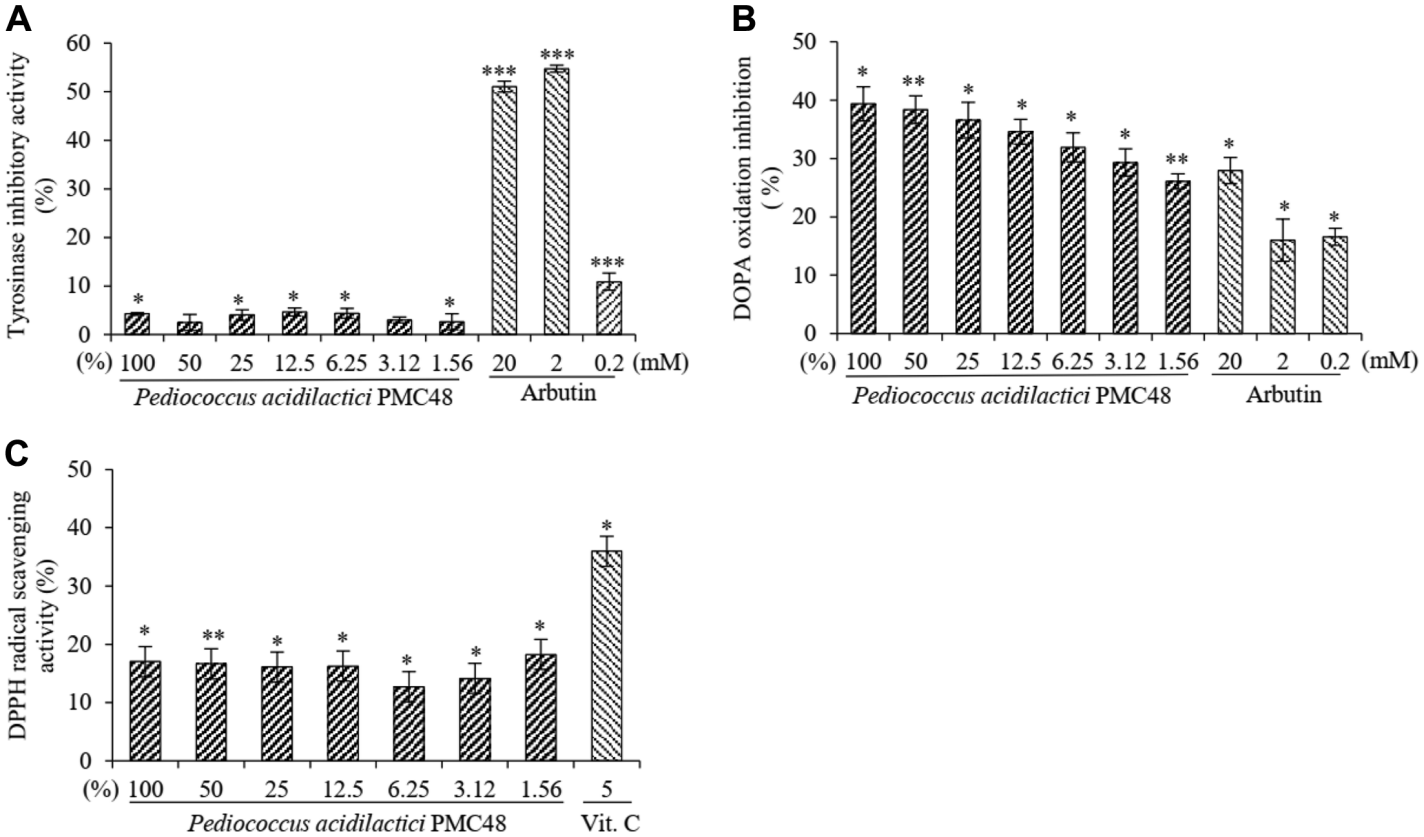
Inhibitory effect of tyrosinase and dopa oxidation of *Pediococcus acidilactici* PMC48 culture filtrate. Arbutin and kojic acid were used as positive standards in the above assay (**A**, **B**). Antioxidative capacity of *P. acidilactici* PMC48 culture filtrate was evaluated by determination of 2,2-diphenyl-1-picryl-hydrazyl scavenging capacity. Vitamin C was used as positive standards in the above assay (**C**). Data are mean ± SD of three separate experiments. Values are significantly different by comparison with the control. *p < 0.05; **p < 0.01; ***p < 0.001.

The main method of treating hypermelanosis so far is by using an agent that can inhibit melanin synthesis in melanocytes [26, 27]. Therefore, we investigated the inhibitory effect of PMC48 on tyrosinase, a melanin synthase, to determine whether this PMC48 strain might be compatible with the existing melanin synthesis technology in addition to its direct melanin degradation effect (Fig. 4). Tyrosinase inhibitory effects of PMC48 strains were measured by comparing two tyrosinase inhibitory tests using tyrosine and 3,4-dihydroxyphenylalanine (L- DOPA) with representative tyrosinase inhibitors hydroquinone and arbutin. In the tyrosinase inhibition test using tyrosine as a substrate, arbutin and hydroquinone had great inhibitory effects, whereas PMC48 culture was ineffective (Fig. 4A). In the tyrosinase inhibition test based on L-DOPA, PMC48 cultures also had inhibitory effects, similar to inhibitory effects of arbutin and hydroquinone (Fig. 4B).

When exposed to ultraviolet radiation, the human skin produces profuse reactive oxygen species (ROS) which in turn activate tyrosinase by mobilizing α-melanocyte-stimulating hormone in the epidermis and finally stimulates melanocytes to produce melanin [28]. In this regard, a strategy for developing agents having both tyrosinase-suppressing and antioxidant effects has recently emerged [28]. Therefore, this study also tested the effect of PMC48 along with vitamin C as a representative antioxidant. Results confirmed that the PMC48 culture solution had DPPH radical scavenging effect of 18.5% (*p* < 0.01) (Fig. 4C). These results suggest that PMC48 strain has a melanin synthesis inhibitory effect by having an antioxidant effect along with a tyrosinase-inhibiting effect when L-DOPA is used as the substrate.

### Whitening Activity of *P. acidilactici* PMC48 in B16F10 Murine Melanoma Cells

**Fig. 5:**
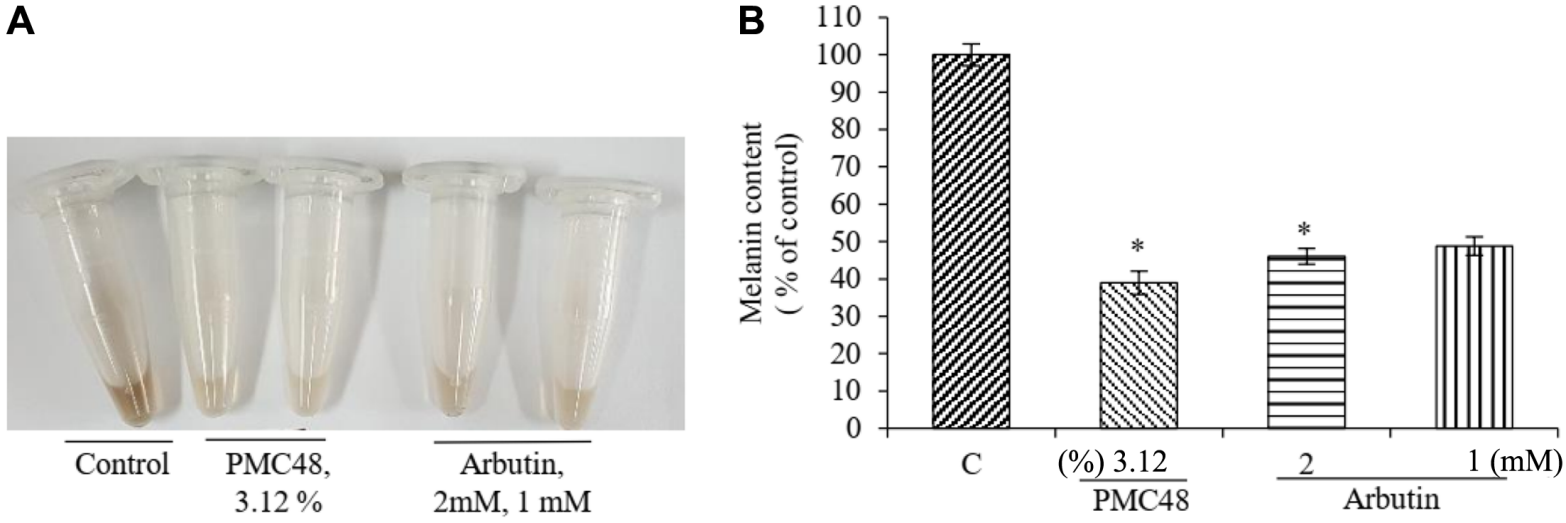
Inhibitory effects of *Pediococcus acidilactici* PMC48 culture filtrate on melanogenesis. B16F10 cells were cultured in the presence or absence of *P. acidilactici* PMC48 for 72 h after treatment with α-melanocyte-stimulating hormone (α-MSH). Cells were harvested in a microcentrifuge tube (**A**), and the optical density was determined (**B**). Results are represented as percentages of control, and the data are presented as mean ± SD of three separate experiments. Values are significantly different by comparison with the control. *p < 0.05; **p < 0.01; ***p < 0.001.

Based on results of in vitro direct melanin-degrading and melanin synthesis-inhibiting effects of PMC48 shown above, its whitening effect on melanocytes was tested (Fig. 5). The whitening effect test of PMC48 strain on melanocytes B16F10 activated by α-melanocyte-stimulating hormone (α-MSH) showed a significant decrease in the amount of melanin (Fig. 5A). This effect of PMC48 culture was quantified with optical absorbance method (Fig. 5B) and enzyme-linked immuno-sorbent assay (ELISA) (Fig. 5C).

### Safety Properties of *P. acidilactici* PMC48

**Fig. 6:**
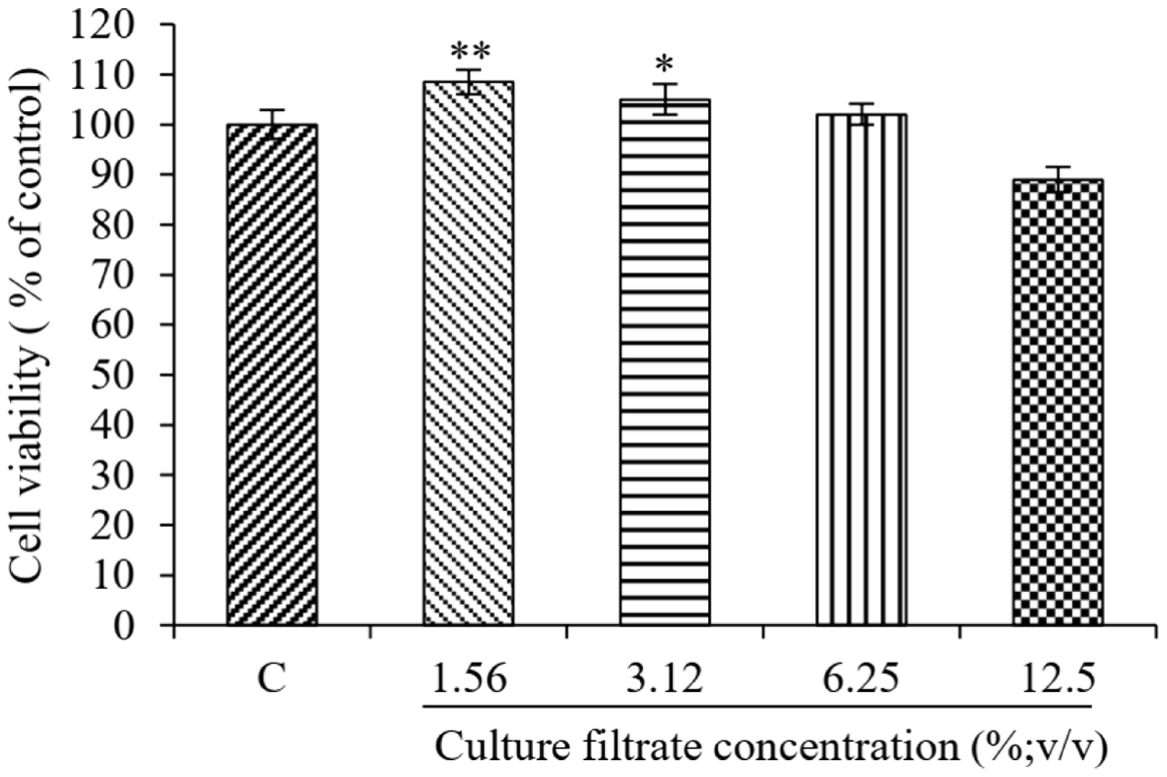
Cell viability assay. B16F10 cells were treated with various concentration of bacterial culture filtrate (1.0, 2.5, 5.0, 7.5, and 10%; v/v) for 24 h and the cell viability was measured by MTT assay. Results are expressed as percentage of cell viability relative to control. Values are significantly different by comparison with the control. *p < 0.05; **p < 0.01; ***p < 0.001.

The safety of the PMC48 strain in the development of hypermelanosis treatment was evaluated. Cytotoxicity experiments using B16F10 cells showed no toxic effects under all conditions (Fig. 6).

In conclusion, PMC48, a new strain of *P. acidilactici*, has melanin synthesis inhibitory effect which is the main mechanism of action of hypermelanosis treatment. It also has direct melanin-degrading effect. In this regard, PMC48 is a promising candidate with high development value as a treatment for hypermelanosis.

## Figures and Tables

**Table 1:** Ability of melanin degrading isolate PMC48 to form acid from different carbohydrates.

No	Type of test	24h	48h	No		Type of test	24h	48h
0	Control	+	+	25		Esculine	+	+
1	Glycerol	-	-	26	-	Salicin	+	+
2	Erythritol	-	-	27		D-Cellibiose	+	+
3	D-arabinose	-	-	28		D-Maltose	+	+
4	L-arabinose	+	+	29		D-Lactose	-	+
5	D-ribose	+	+	30		D-Melibiose	-	-
6	D-xylose	+	+	31		D-Sacharose	+	+
7	L-xylose	-	-	32		D-Trehalose	+	+
8	D-adonitel	-	-	33		Inulin	-	-
9	Methyl-βD-xylopyranoside	-	-	34		D-Melezitose	-	-
10	D-galactose	+	+	35		D-Raffinose	-	-
11	D-glucose	+	+	36		Amidon	-	-
12	D-fructose	+	+	37		Glycogen	-	-
13	D-mannose	+	+	38		Xylitol	-	-
14	L-sorbose	-	-	39		Gentibiose	+	+
15	L-rhamnose	-	-	40		D-Turanose	-	-
16	Dulcitol	-	-	41		D-Lyxose	-	-
17	Inocitol	-	-	42		D-Tagatose	+	+
18	D-mannitol	-	+	43		D-Fucose	-	-
19	D-sorbitol	-	+	44		L-Fucose	-	-
20	Methyl-αD-mannopyranoside	-	-	45		D-arabitol	-	-
21	Methyl-αD-glucopyranoside	-	-	46		L-arabitol	-	-
22	N-acetylglucosamine	+	+	47		Potassium gluconate	-	+
23	Amygdaline	+	+	48		Potassium 2 ketogluconate	-	-
24	Arbutine	+	+	49		Potassium 5 ketogluconate	-	-

(+): positive reaction (yellow), no. 25 (black); (-): negative reaction (violet)

**Table 2:** Identification of isolated bacterial strain, PMC48, based on 16S rRNA gene sequence analysis and their close relative published in DNA databases.

NCBI Reference	Organism	Length	Score	Identities	Gaps	E Value
NR_042057.1	*Pediococcus acidilactici* DSM 20284	1569	2732 bits (1479)	1497/1505 (99%)	4/1505 (0%)	0.0
NR_042058.1	*Pediococcus pentosaceus* DSM 20336	1569	2632 bits (1425)	1481/1508 (98%)	4/1508 (0%)	0.0
NR_041640.1	*Pediococcus acidilactici* NGRI 0510Q	1540	2573 bits (1393)	1450/1474 (98%)	18/1474 (1%)	0.0
NR_042401.1	*Pediococcus stilesii* strain FAIR-E 180	1529	2571 bits (1392)	1461/1496 (98%)	2/1496 (0%)	0.0
NR_075029.1	*Pediococcus claussenii* strain ATCC BAA-344	1567	2518 bits (1363)	1460/1507 (97%)	6/1507 (0%)	0.0
NR_042623.1	*Pediococcus argentinicus* strain CRL 776	1492	2484 bits (1345)	1445/1494 (97%)	6/1494 (0%)	0.0
NR_042232.1	*Pediococcus claussenii* strain P06	1472	2423 bits (1312)	1419/1471 (96%)	5/1471 (0%)	0.0
NR_113922.1	*Pediococcus parvulus* strain NBRC 100673	1501	2386 bits (1292)	1429/1496 (96%)	7/1496 (0%)	0.0
NR_043290.1	*Pediococcus cellicola* strain Z-8	1542	2377 bits (1287)	1432/1503 (95%)	5/1503 (0%)	0.0
NR_025388.1	*Pediococcus inopinatus* strain DSM 20285	1551	2366 bits (1281)	1430/1503 (95%)	5/1503 (0%)	0.0
NR_042087.1	*Pediococcus damnosus* strain DSM 20331	1561	2351 bits (1273)	1428/1503 (95%)	10/1503 (1%)	0.0
NR_043291.2	*Pediococcus ethanolidurans* strain Z-9	1501	2344 bits (1269)	1404/1470 (96%)	6/1470 (0%)	0.0
NR_029136.1	*Pediococcus parvulus* strain S-182	1436	2302 bits (1246)	1374/1437 (96%)	6/1437 (0%)	0.0
NR_125575.1	*Lactobacillus brantae* DSM 23927 strain SL1108	1545	2289 bits (1239)	1417/1502 (94%)	16/1502 (1%)	0.0
NR_115654.1	*Pediococcus damnosus* strain JCM 5886	1497	2281 bits (1235)	1411/1499 (94%)	11/1499 (1%)	0.0
NR_109538.1	*Lactobacillus curieae* strain S1L19	1540	2265 bits (1226)	1417/1510 (94%)	9/1510 (1%)	0.0
NR_113290.1	*Lactobacillus senioris* DSM 24302 = JCM 17472 strain YIT 12364	1562	2265 bits (1226)	1416/1509 (94%)	8/1509 (1%)	0.0
NR_113289.1	*Lactobacillus saniviri* JCM 17471 = DSM 24301	1558	2263 bits (1225)	1420/1513 (94%)	18/1513 (1%)	0.0
NR_116411.1	*Lactobacillus kimchicus* JCM 15530 strain DCY51	1499	2235 bits (1210)	1409/1504 (94%)	17/1504 (1%)	0.0
NR_042442.1	*Lactobacillus malefermentans* strain DSM 5705	1556	2233 bits (1209)	1410/1506 (94%)	17/1506 (1%)	0.0


**Table 3:** Comparison of the chromosomal properties of *Pediococcus acidilactici* strains.

Strain	PMC48	LPBC161	S1	MA18/5M	K3	NGRI 0510Q^T^
Sources	Sesame leaf kimchi	Mature coffee cherry	Makgeolli	Pasture Gramineae	Nuruk	Ryegrass Silage
Genome size (bp)	2,043,929	1,960,506	1,980,172	1,992,928	1,991,399	2,047,078
G+C content (%)	42.2	42.2	42	42.1	42.1	41.2
Predicted CDS	2,026	2,019	1,525	1,967	1,525	2,154
Number of rRNA genes	15	6	7	NC	8	2
Number of tRNA genes	57	52	40	NC	50	54

NC: not confirmed.

## References

[1] Sarangarajan R, Apte SP (2006). The polymerization of melanin: a poorly understood phenomenon with egregious biological implications. Melanoma Res..

[2] Riley PA (1997). Melanin. Int. J. Biochem. Cell Biol..

[3] Matts PJ, Dykes PJ, Marks R (2007). The distribution of melanin in skin determined in vivo. Br. J. Dermatol..

[4] Costin GE, Hearing VJ (2007). Human skin pigmentation: melanocytes modulate skin color in response to stress. FASEB J..

[5] Brenner M, Hearing VJ (2008). The protective role of melanin against UV damage in human skin. Photochem. Photobiol..

[6] Ortonne JP, Passeron T (2005). Melanin pigmentary disorders: treatment update. Dermatol. Clin..

[7] Gimenez Garcia RM, Carrasco Molina S (2019). Drug-Induced hyperpigmentation: Review and case series. J. Am. Board Fam. Med..

[8] Rendon M, Berneburg M, Arellano I, Picardo M (2006). Treatment of melasma. J. Am. Acad. Dermatol..

[9] Rendon MI (2019). Hyperpigmentation disorders in hispanic population in the United States. J. Drugs Dermatol..

[10] Mohorčič M, Friedrich J, Renimel I, André P, Mandin D, Chaumont J-P (2007). Production of melanin bleaching enzyme of fungal origin and its application in cosmetics. Biotechnol. Bioprocess Eng..

[11] Kim BS, Blaghen M, Hong HS, Lee KM (2016). Purification and characterization of a melanin biodegradation enzyme from Geotrichum sp. Int. J. Cosmet. Sci..

[12] Luther JP, Lipke H (1980). Degradation of melanin by *Aspergillus fumigatus*. Appl. Environ. Microbiol..

[13] Awong-Taylor J, Craven KS, Griffiths L, Bass C, Muscarella M (2008). Comparison of biochemical and molecular methods for the identification of bacterial isolates associated with failed loggerhead sea turtle eggs. J. Appl. Microbiol..

[14] Nguyen MH, Sigoillot JC (1996). Isolation from coastal sea water and characterization of bacterial strains involved in non-ionic surfactant degradation. Biodegradation.

[15] Naz T, Khan MD, Ahmed I, Rehman SU, Rha ES, Malook I (2016). Biosorption of heavy metals by *Pseudomonas* species isolated from sugar industry. Toxicol. Ind. Health.

[16] Janda JM, Abbott SL (2007). 16S rRNA gene sequencing for bacterial identification in the diagnostic laboratory: pluses, perils, and pitfalls. J. Clin. Microbiol..

[17] Kim M, Chun J (2014). 16S rRNA Gene-Based Identification of Bacteria and Archaea using the EzTaxon Server.

[18] Tatusov RL, Galperin MY, Natale DA, Koonin EV (2000). The COG database: a tool for genome-scale analysis of protein functions and evolution. Nucleic Acids Res..

[19] Lee I, Ouk Kim Y, Park SC, Chun J (2016). OrthoANI: An improved algorithm and software for calculating average nucleotide identity. Int. J. Syst. Evol. Microbiol..

[20] Aubin GG, Bemer P, Kambarev S, Patel S, Lemenand O, Caillon J (2016). *Propionibacterium namnetense* sp. nov., isolated from a human bone infection. Int. J. Syst. Evol. Microbiol..

[21] Muynarsk ESM, de Melo Pereira GV, Mesa D, Thomaz-Soccol V, Carvalho JC, Pagnoncelli MGB (2019). Draft genome sequence of *pediococcus acidilactici* strain LPBC161, isolated from mature coffee cherries during natural fermentation. Microbiol. Resour. Announc..

[22] Park GS, Hong SJ, Jung BK, Park S, Jin H, Lee SJ (2017). Whole genome sequence of lactic acid bacterium Pediococcus acidilactici strain S1. Braz J. Microbiol..

[23] Barreau G, Tompkins TA, de Carvalho VG (2012). Draft genome sequence of probiotic strain Pediococcus acidilactici MA18/5M. J. Bacteriol..

[24] Park GS, Hong SJ, Park S, Jin H, Lee SJ, Shin JH (2017). Draft genome sequence of alcohol-tolerant bacteria Pediococcus acidilactici strain K3. Braz. J. Microbiol..

[25] Doi K, Mori K, Tashiro K, Fujino Y, Nagayoshi Y, Hayashi Y (2013). Draft Genome Sequence of *Pediococcus lolii* NGRI 0510Q(T) Isolated from Ryegrass Silage. Genome Announce..

[26] Nihei K, Kubo I (2003). Identification of oxidation product of arbutin in mushroom tyrosinase assay system. Bioorg. Med. Chem Lett..

[27] Palumbo A, d'Ischia M, Misuraca G, Prota G (1991). Mechanism of inhibition of melanogenesis by hydroquinone. Biochim. Biophys Acta.

[28] Wang Y, Hao MM, Sun Y, Wang LF, Wang H, Zhang YJ (2018). Synergistic promotion on tyrosinase inhibition by antioxidants. Molecules.

[29] Bilodeau ML, Greulich JD, Hullinger RL, Bertolotto C, Ballotti R, Andrisani OM (2001). BMP-2 stimulates tyrosinase gene expression and melanogenesis in differentiated melanocytes. Pigment Cell Res..

[30] Tada H, Shiho O, Kuroshima K, Koyama M, Tsukamoto K (1986). An improved colorimetric assay for interleukin 2. J. Immunol Method.

